# COVID-19 PANDEMIC AND RESILIENCE IN STAFF OF A CONTINUING CARE RETIREMENT COMMUNITY

**DOI:** 10.1093/geroni/igac059.1622

**Published:** 2022-12-20

**Authors:** Zachary Hathaway, Kathleen Fisher, Martha Coates, Shelby Hufnal, Isabella Stoll, Justine Sefcik, Rose Ann DiMaria-Ghalili

**Affiliations:** Drexel University, Philadelphia, Pennsylvania, United States; Drexel University, Philadelphia, Pennsylvania, United States; Drexel University, Philadelphia, Pennsylvania, United States; Drexel University, Philadelphia, Pennsylvania, United States; Drexel University, Philadelphia, Pennsylvania, United States; Drexel University, Philadelphia, Pennsylvania, United States; Drexel University, College of Nursing and Health Professions, Philadelphia, Pennsylvania, United States

## Abstract

Residents and staff of continuing care retirement communities (CCRC) experienced many challenges during the COVID-19 pandemic including loss, social isolation, and staff turnover. This study examined factors that contribute to resilience in staff during the late stage of the pandemic using the Connor-Davidson Resilience Scale. Resilience scores ranged from 0 (low) to 100 (high). A total of 96 staff (76% female) were enrolled, and average age was 48.41 years (SD = 16.16). Average resilience in staff was 75.16 (SD = 11.81). Those under 35 years of age reported lower resilience scores (M = 67.38) compared to those 35-49 years of age (M = 76.65), 50-64 (M = 75.83), and 65 years and older (M = 82.71), p <.05. Staff who were married scored higher than those who were not (M = 76.63 vs 69.05), p < .05. Findings can inform professional development programs aimed at increasing coping skills in staff.

